# A High-Density Genetic Linkage Map and QTL Mapping for Sex in Black Tiger Shrimp (*Penaeus monodon*)

**DOI:** 10.3389/fgene.2019.00326

**Published:** 2019-04-09

**Authors:** Liang Guo, Yu-Hui Xu, Nan Zhang, Fa-Lin Zhou, Jian-Hua Huang, Bao-Suo Liu, Shi-Gui Jiang, Dian-Chang Zhang

**Affiliations:** ^1^Key Laboratory of South China Sea Fishery Resources Exploitation and Utilization, Ministry of Agriculture and Rural Affairs, South China Sea Fisheries Research Institute, Chinese Academy of Fishery Sciences, Guangzhou, China; ^2^Guangdong Provincial Engineer Technology Research Center of Marine Biological Seed Industry, Guangzhou, China; ^3^Biomarker Technologies Corporation, Beijing, China

**Keywords:** *Penaeus monodon*, genetic linkage map, sex, QTL mapping, RADseq

## Abstract

The black tiger shrimp, *Penaeus monodon*, is important in both fishery and aquaculture and is the second-most widely cultured shrimp species in the world. However, the current strains cannot meet the market needs in various cultural environments, and the genome resources for *P. monodon* are still lacking. Restriction-site associated DNA sequencing (RADseq) has been widely used in genetic linkage map construction and in quantitative trait loci (QTL) mapping. We constructed a high-density genetic linkage map with RADseq in a full-sib family. This map contained 6524 single nucleotide polymorphisms (SNPs) and 2208 unique loci. The total length was 3275.4 cM, and the genetic distance was estimated to be 1.1 Mb/cM. The sex trait is a dichotomous phenotype, and the same interval was detected as a QTL using QTL mapping and genome-wide association analysis. The most significant locus explained 77.4% of the phenotype variance. The sex locus was speculated to be the same in this species based on the sequence alignments in Mozambique, India, and Hawaii populations. The constructed genetic linkage map provided a valuable resource for QTL mapping, genome assembly, and genome comparison for shrimp. The demonstrated common sex locus is a step closer to locating the underlying gene.

## Introduction

The black tiger shrimp, *Penaeus monodon*, is naturally distributed in the Indo-West Pacific region and is cultured in much of this region (Motoh, 1985). It is commercially important, both in capture fisheries and in aquaculture (Brackishwater Aquaculture Information System, 1988; FAO, 2018), and is the second-most widely cultured shrimp species only after Pacific white shrimp (FAO, 2018). Substantial efforts have been made to improve the quality of the breeding strains. In China, the strains “Nanhai No. 1” and “Nanhai No. 2” are aimed for growth, and the survival rates are improving through selective breeding and cross-breeding. In India, breeding for disease resistance has been performed (Robinson et al., 2014). Even so, the cultured strains still cannot meet the market needs, especially during severe disease outbreak and negative influences to aquaculture production expansion (Robinson et al., 2014; FAO, 2018). Thus, it is necessary to develop genome resources for breeding to achieve sustainable aquaculture.

This species contains 44 chromosomes that is based on the karyotype (Kong, 1993; You et al., 2010). The different kind of markers, including amplified fragment length polymorphism (AFLP), simple sequence repeat (SSR), and single nucleotide polymorphism (SNP), were used to construct the genetic linkage maps for this species (Tassanakajon et al., 2002; Wilson et al., 2002; Wuthisuthimethavee et al., 2005; You et al., 2010; Baranski et al., 2014; Robinson et al., 2014). The highest density map was constructed with 3959 coding SNPs (cSNPs) that were genotyped by an Illumina iSelect genotyping array, which contains 2170 unique loci, and the flanking sequences have also been released (Baranski et al., 2014). This map was constructed using samples that were collected from coastal of India, so it is referred to as the India map in this study. Recently, restriction-site associated DNA sequencing (RADseq) has been widely used to construct high-density genetic linkage maps (Robledo et al., 2018), including that of the Pacific white shrimp (Yu et al., 2015) and Kuruma prawn (Lu et al., 2016). With the advantage of RADseq, a high-density genetic linkage map could be easily achieved, which is important for locating the functional genes underlying the traits, assembling the genome sequences, and comparing chromosomal evolution (Zhao et al., 2013). For traits that are related to growth (Sraphet, 2004) and disease resistance (Robinson et al., 2014), quantitative trait loci (QTL) mapping has been performed in this species. These studies could provide clues in genome dissecting and marker-assisted breeding.

The mechanism of sex determination is diverse, especially for the master determining gene. The master determining genes have been confirmed in several fish (Kikuchi and Hamaguchi, 2013) and insects (Geuverink and Beukeboom, 2014). The progress in Decapoda lags behind, despite its high economic importance (Chandler et al., 2017). The black tiger shrimp is gonochoristic; the female reaches a relatively large size, and size dimorphism appears in the late development stage (Primavera et al., 1998). Thus, focusing on sex determination could deepen the understanding of the mechanism of sex determination in invertebrate and facilitate the potential usage in production (Martínez et al., 2014). The heteromorphic sex chromosomes have not been observed (You et al., 2010), which may hint that the sex chromosomes are at the initial stage (Sember et al., 2018). In such case, the sex QTL are detected mainly through QTL mapping, such as in common carps (Peng et al., 2016; Feng et al., 2018), yellow drum (Qiu et al., 2018), Nile tilapia (Eshel et al., 2012; Palaiokostas et al., 2013), and the Tiger Pufferfish (Kamiya et al., 2012). Two independent studies, one using AFLP (Staelens et al., 2008) and the other using cSNPs (Robinson et al., 2014), located only one sex QTL in the black tiger shrimp, which demonstrates that the sex is mainly determined by only one genetic factor (Martínez et al., 2014). Even though these two studies published a closely linked sex segment and genetic map-associated sequences, respectively, the reported two sex loci could not corroborate each other because they lacked the common sequences to anchor the markers. As known, the former sex-linked segment is from Moana Technologies in Hawaii, and the latter is from the Indian population. The study on the genetic analysis of the black tiger shrimp showed significant genetic distinctions in individuals that reside at the peripheries of the Indian and Pacific Ocean distribution range, which is supported by the result of microsatellites and mtDNA. The population from the Pacific Ocean is differentiated from the population from the Indian Ocean. The India and Mozambique populations were statistically significantly differentiated (Fst = 0.065, *p*-value < 0.05) (Waqairatu et al., 2012). It was reported that the master sex-determining gene may vary in different strains or populations in insects (Biedler and Tu, 2016) and fish (Wilson et al., 2014), and there is no report on the variation in master sex-determining genes in shrimp from differentiated populations.

To deepen the knowledge in sex determination of black tiger shrimp, we conducted this research. First, we constructed a high-density genetic linkage map using RADseq and located the sex QTL using QTL mapping and a family-based genome-wide association study (GWAS) in the Mozambique population. We only detected one sex QTL, which is consistent with the conclusion that the sex is mainly determined by only one genetic factor, and the segregation pattern supports the WZ–ZZ chromosomal system. Moreover, we compared the published sex-linked segment, the India sex QTL and the Mozambique sex QTL. All three loci were located in the same region, which hints that the sex determination region of black tiger shrimp in differential populations may be the same.

## Materials and Methods

### Ethics Statement

All experiments in this study were approved by the Animal Care and Use Committee of South China Sea fisheries Research Institute, Chinese Academy of Fishery Sciences, and were performed according to the regulations and guidelines that were established by this committee.

### Sample Collection

The full-sib black tiger shrimp family that was used for QTL mapping was an F2 population. The F0 population was collected from the Mozambique Channel with the permission obtained in accordance with the national guidelines and cultured in the Shenzhen Experiment Base of the South China Sea Fisheries Research Institute. The shrimps from the first filial generations were artificially inseminated and tagged. One full-sib family, including the F1 parents and the F2 offspring, was randomly selected for genetic linkage map construction and QTL mapping. Broodstock mating, culturing, and larval rearing have been described previously (Sun et al., 2015). At approximately 60 days, the growth traits, including carapace length (CL), body length (BL), body weight (BW), and sex were recorded according to previous descriptions (Motoh, 1985; Sun et al., 2015), and abdominal muscles from the two parents and offspring were preserved in 95% alcohol for genotyping. Assessing the influence of sex on the growth traits (Kruskal–Wallis test) was performed using Minitab 17^1^.

### High-Throughput Sequencing and Genetic Linkage Map Construction

The method SLAF-seq (Sun et al., 2013) was used to survey the genome. The enzyme EcoRI (G|AATTC), NlaIII (CATG|), and MseI (T|TAA) and the effective library length of 380–430 bp were manipulated to enrich the sequences, and the read pairs with read length of 100 bp were sequenced on the platform Illumina HiSeq 2500 system (Illumina, Inc., San Diego, CA, United States). The procedures of library construction and sequencing were conducted in Biomarker Technologies Corporation (Beijing, China).

**FIGURE 1:**
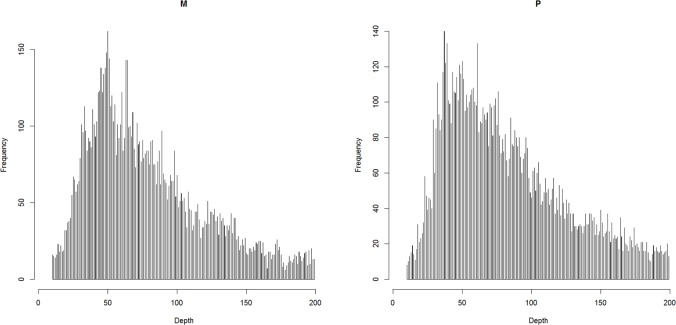
The coverage in parents after filtering out the reads that over-mapped and over-separated.

**FIGURE 2:**
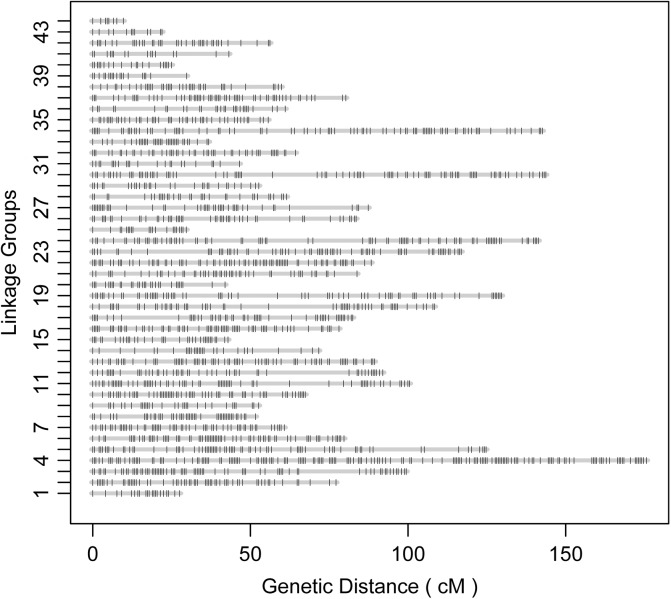
Illustration of the constructed genetic linkage map.

The raw reads were checked using FastQC v0.11.5 (Andrews, 2015) and were filtered using Trimmomatic v0.36 (Bolger et al., 2014). The reads were first trimmed to be 50 bp in length, and then the bases with a quality lower than 20 were cut off at the start and at the end. The reads were also scanned with four-base wide sliding window and average base quality threshold of 15. Finally, reads shorter than 40 bp were dropped off. The clean reads were mapped to the reference assembly (unpublished) with BWA-backtrack (Li and Durbin, 2009). The reference assembly was assembled with short reads from Illumina platform, the contig N50 and scaffold N50 were 10 and 383 kbp, respectively. The properly mapped primary read pairs with insert size range of 100–700 bp were selected for next step. The alignments were piled up with a minimum base quality of 20 and a minimum map quality of 20 with SAMtools v1.5 (Li et al., 2009). The genetic map was constructed with Lep-MAP3 (Rastas, 2017), in which the genotype likelihoods were calculated with the script pileup2posterior.awk according to the description for RADseq (Yang et al., 2016). The markers were filtered as below: (1) coverage in parents should be between 10 and 200 based on the expected normal distribution (Davey et al., 2013) (Figure 1); (2) the number of missing individuals should be no more than 10; and (3) the inheritance should be in agreement with Mendel’s law of segregation (*p*-value = 0.01). The parentage relationship was checked by calculating identity by decent. The linkage groups were assigned with an LOD score limit of 15 and a minimum marker number of 9 using informative markers in both parents. Other singular markers were assigned with an LOD score limit of 5. The marker order in each linkage group was obtained with best score from 10 independent runs. The genetic distance was converted by using the Kosambi mapping function.

The average interval was calculated as the quotient that divided the length of the accumulated linkage groups by the difference of the number of loci minus the number of linkage groups (Ren et al., 2016). The expected genome length was calculated as the sum of length of each linkage group, which was the sum of two times the average interval, and the length of itself (Fishman et al., 2001). The genome coverage was estimated as the quotient that divided the expected genome length by the accumulated map length.

### Sex QTL Detection

The phased genotypic data were exported from Lep-MAP3 (Rastas, 2017). The QTL were detected with MapQTL 6 (Ooijen et al., 2009). The potential QTL were first detected using internal mapping, and the threshold of the significant level of the LOD score was determined using a permutation test with a *P*-value of 0.05 and with 10,000 permutations. Then, the SNPs closest to the significant QTL were taken as cofactors to narrow the interval in subsequent MQM mapping. As an alternative method of QTL detection, an association analysis was performed using the GWAF package (Chen and Yang, 2010), which was designed for family data. The genotypes of the filtered SNPs were directly transformed to fit this program. The kinship was calculated according to the pedigree. The association between sex and genotypes was performed using logistic regression (Chen and Yang, 2010). Bonferroni correction was performed to control the false-positive rate. The candidate genes and the annotation from the sex QTL interval were obtained from our genome program (unpublished).

### Sex Loci Comparison

The relation between the sex-linked segment (Staelens et al., 2008), the India sex QTL, and Mozambique sex QTL was explored. The consensus between the sex-linked segment, India sex QTL, and Mozambique sex QTL was assessed. The mRNA sequences among the India sex QTL intervals were downloaded and concentrated according to the order on the India map (Robinson et al., 2014). The scaffolds among the Mozambique sex QTL intervals in this study were also concentrated according to their position on the Mozambique map. The synteny was constructed using the LAST program (Kielbasa et al., 2011), which can find homologous sequences that take the feature of the reverse complement and large gap into consideration. The sex-linked segment (Staelens et al., 2008) was treated as a query to search for the genome assembly using BLAST (Altschul et al., 1997).

### QTL Validation

The significant sites in the Mozambique sex QTL interval were validated in another population. One hundred individuals were randomly collected from our breeding population, which is an admixture population. The DNA was extracted using a HiPure Tissue and Blood DNA Kit (Magen, Guangzhou, China), and the quality was tested with 1% agarose gel electrophoresis. Primers were designed using Primer-BLAST to cover the SNPs. PCRs were performed using a PCR amplification Kit (PrimeSTAR^®^HS, Takara, Dalian, China) with a program of 5 min at 94°C, 35 cycles of 45 s at 94°C, 45 s at 60°C, 45 s at 72°C, and 10 min at 72°C. Finally, PCR products were genotyped and sequenced on a 3130xl capillary DNA analyzer (Applied Biosystems, Foster City, CA, United States), and the allele sizes were analyzed using GeneMapper version 4.0 (Applied Biosystems, Foster City, CA, United States) and sequences were viewed using the Seqman software package (Lasergene Version 7.1; DNA Star Inc., Madison, WI, United States). The sites that contained the target SNPs in the mapping population were genotyped in the validation population as SNP or Indel (insertion or deletion). A genotypic (2 df) test was performed to test the relationship between genotype and sex (Purcell et al., 2007).

## Results

### Phenotyping

A full-sib family, two parents, and 98 offspring Supplementary Table S1) were sampled. The traits of CL, BL, and BW were 20.1 ± 2.9 mm, 71.1 ± 9.3 mm, and 5.1 ± 1.7 g (mean ± SD), respectively, and only BW fit to the normal distribution (p value > 0.05). These three traits were significantly related with each other (*p*-value = 0.00), and Pearson’s correlation coefficients were 0.83, 0.83, and 0.95 for the trait pairs CL–BL, CL–BW, and BL–BW, respectively. In the offspring, there were 52 males and 46 females. There was no significant difference in these three growth traits between the different sexes (*p*-value > 0.05).

### Genetic Linkage Map Construction

After filtering, 93.61% of the base had a quality above 30. Each offspring obtained 680 ± 395 (mean ± SD) thousand read pairs, one parent obtained 20 million read pairs and the other parent obtained 18 million read pairs. Only 64.0% were primarily and properly mapped, among which only 47.8% were used in later steps, with a mapping quality above 20. After strict filtering, 6821 SNPs were selected to construct the genetic map.

According to the relatedness based on markers, all the offspring were assigned to the targeted family. The sex-averaged consensus genetic map was constructed, and 6524 SNPs that were located on 2354 scaffolds (Supplementary Tables S2, S3) were assigned into 44 linkage groups (Figure 2 and Table 1), which contained 2208 unique loci. The consensus map was 3275.4 cM in length. The average interval between loci was 1.51 cM. This map was estimated covering 96.1% of the genome. Based on that, the genome size was 2.47 Gb (C-value: 2.53) (Gregory, 2018), the genetic distance was estimated as 1.1 Mb/cM. The length of each linkage group ranged from 9.7 to 175.0 cM, and the number of unique loci varied from 8 to 161. The scaffolds of the genome assembly were anchored to the genetic linkage map. After filtering the scaffolds those were anchored to more than one linkage group and supported with only one SNP, 3202 SNPs left on the genetic linkage map.

**Table 1:** Summary of the genetic linkage map.

Item	Content
No. of individuals	98 offspring and two parents
No. of linkage groups	44
No. of markers	6524
No. of unique markers	2208
Summary unique marker number/LG	Minimum = 8, mean = 50, maximum = 161
Average LG length (cM)	Minimum = 9.7, mean = 74.4, maximum = 175.6

### Sex QTL Detection and Validation

**FIGURE 3:**
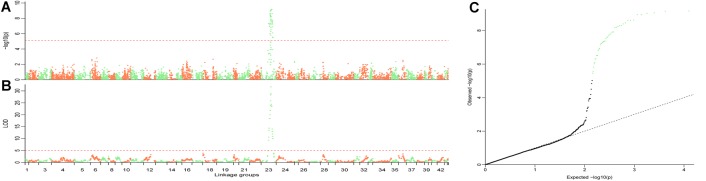
Illustration of the QTL for trait sex. GWAS **(A)** and QTL mapping **(B)** were performed to locate the sex QTL. The overlapped interval in these two methods demonstrates that this genome-wide significant locus is the only one interval in which the sex QTL was located. The family structure always confuses the result in GWAS. QQplot **(C)** for the GWAS shows that the result is statistically significant with an efficient family structure correction. The length of each linkage groups **(A,B)** is plotted as the genetic distance in *x*-axis. The genome-wide significant points in **C** are highlighted in light green.

The QTL were detected using the methods of QTL mapping and GWAS. For the trait sex, only one locus on group 23 was genome-wide significant in both methods, and these two intervals overlapped (Figure 3). The LOD score of 5.1 and the *p*-value 7.66 × 10^−6^ were calculated as the genome-wide significant threshold in QTL mapping and GWAS, respectively. The feature of this QTL in QTL mapping is described below. The most related loci explained 77.4% phenotype variance. This interval (the LOD score larger than the threshold) ranged from 53.49 to 92.45 cM on linkage group 23, and the peak was located at 74.45 cM. MQM confirmed that this interval contained only one QTL. To confirm the result, primers were designed to validate these sites in another randomly collected breeding population. Finally, five sites were successfully genotyped and were significant associated with sex, including X262, X881, X5302, X5303, and X748, with *p*-values of 2.49 × 10^−19^, 1.30 × 10^−10^, 1.11 × 10^−12^, 2.83 × 10^−12^, and 7.99 × 10^−8^, respectively (Table 2).

**Table 2:** The sites located in the sex QTL interval and successfully genotyped in the validation population.

Site	Position (cM)	Primers (forward/reverse, 5′- > 3′)	Genotypes	Female	Male	*P*-value	Marker type^a^
X5303	69.34	ACTGTCGTTACACGGATTGGA/ATTATGGGAGACACCGCTTAC	AA:GG:GA	22:5:13	1:40:6	2.83 × 10^−12^	SNP
X748	69.34	GACCCACAGACCATTTCACAG/TCAATCTAACCCACCATCTACCT	CC:TT:CT	43:5:0	13:25:11	7.99 × 10^−8^	SNP
X5302	71.38	ACTGTCGTTACACGGATTGGA/ATTATGGGAGACACCGCTTAC	AA:GG:AG:	26:6:17	1:42:5	1.11 × 10^−12^	SNP
X262	74.45	GTGAGCTATTCCACAAAACTTGG/AAAAGGGCAAACAGGTGAGAC	AA:AB	50:0	9:49	2.49 × 10^−19^	Indel A:215 bp, B:220 bp
X881	85.17	CATTGTTTCCCCTCTTTCTTTCA/GAGTAAACCAGCGAGTGAGCG	AA:AB	41:9	9:41	1.30 × 10^−10^	Indel A:309 bp, B:307 bp

*^a^Indels exist beside the sites X262 and X881, the genotypes are showed as amplicon size. These two sites were also confirmed by consensus sequence by direct sequencing.*

**FIGURE 4:**
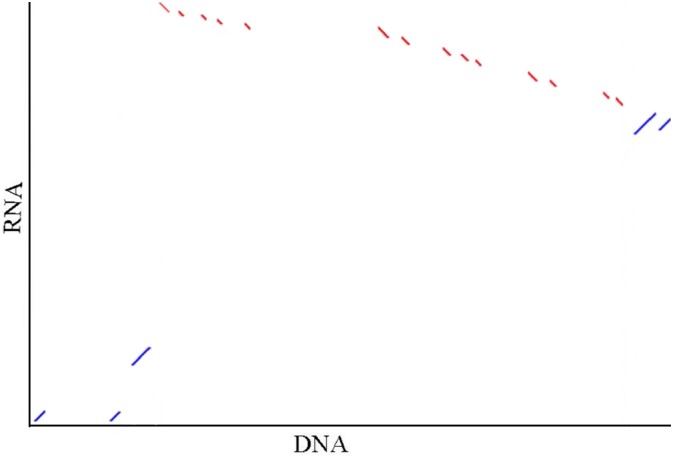
Illustration of the synteny of concentrated sequences from two sex QTL intervals. In the graph, DNA indicates the concentrated DNA sequence (20.6 Mbp) from the Mozambique sex QTL interval and RNA indicates the concentrated RNA sequence (19.6 kbp) from the India sex QTL interval in the previous independent research [4]. Five (accession no. JR222814.1, JR226900.1, JR221289.1, JR198397.1, and JR221656.1) of 17 mRNA are matched to the contigs. The red and blue dots show the alignments are in the reverse direction and in the same direction, respectively. The synteny between the sequences that are located in the same interval from the two independent studies hints that the sex determination region of black tiger shrimp in differential populations may be the same.

Seventeen mRNA sequences (19.6 kbp) and 42 scaffolds (20.6 Mbp) located in the India and Mozambique sex QTL interval were concentrated, respectively. They were confirmed to be of the same locus, with four common gene segments detected based on the synteny (Figure 4). The sex-linked segment from the Hawaii population hit the scaffold 000006388 with an *e*-value of 1 × 10^−130^. This scaffold was anchored at 75.47 cM in the Mozambique sex QTL. In the sex QTL interval, 29 genes (Supplementary Table S4) were located on the scaffolds that contain the SNPs with the highest LOD in QTL mapping.

## Discussion

The black tiger shrimp is an important species in aquaculture and in fishery. In this study, we constructed a genetic linkage map using RADseq and preliminarily located the sex QTL.

The genome assembly is the foundation of structural and functional genomics. With the widely application of next generation sequencing technology, the genome assemblies and genetic linkage maps have rapidly accumulated (Lehmann et al., 2018; Robledo et al., 2018). In fish, 27 chromosome-scale genome assemblies have been published and N50 of the contigs in Nile tilapia, orange clownfish, and Asian seabass is over 1 Mbp (Lehmann et al., 2018). However, the disadvantage of short read blocks is the usage in a complex genome with a large size and a high amount of repetitive sequences. For example, the estimated repetitive sequences account for 79.37% (Yu et al., 2015) and the *C*-value is 2.50 (Chow, 1990) in Pacific white shrimp. The published genome assemblies in shrimp, including cherry shrimp (Kenny et al., 2014), Pacific white shrimp (Yu et al., 2015), Kuruma prawn, and black tiger shrimp (Yuan et al., 2018), assembled using short reads are far from completion compared with those of fish (Lehmann et al., 2018). The N50 of the scaffold/contig for these four genome assemblies are all less than 1 kbp (Kenny et al., 2014; Yu et al., 2015; Yuan et al., 2018). In our study, the quality of the used genome assembly is at a comparable level with the published shrimp genome assemblies. Only approximately 60% of the reads from RADseq were properly mapped to the genome assembly, which was presumed to be mainly caused by the poor quality of genome assembly and the large amount of repetitive sequences. The incompleteness makes fine mapping and genome comparison difficult. Fortunately, longer read sequence technology has been used, such as PacBio SMRT technology. The combination of these technologies is expected to improve the quality of the complex genome assembly (Lehmann et al., 2018).

A genetic linkage map is useful in genome assembly, genome comparison, and QTL mapping. The marker number on the map is determined primarily by the technology and secondarily by the experiment operation. With the advantage of RADseq, the recently published maps contain much more SNPs (Peng et al., 2016); for example, the map for blunt snout bream contains 14,648 SNPs (Wan et al., 2017), and the map for Pacific White Shrimp contains 6359 markers. In our study, the number of unique loci was 2208, which is smaller than the 4693 markers on the Pacific White Shrimp map (Yu et al., 2015). The fewer number of unique loci on our map compared with that of the Pacific White Shrimp map is supposed to be caused by less data input for each individual, fewer offspring, and shorter reads. Compared with the Pacific White Shrimp, the data volume and number of offspring are both approximate by half. Compared with the large number of SNPs, the number of individuals on the recently published map was relatively small. In the blunt snout bream map, the number of unique loci was 5676, which means that every three SNPs were located on the same unique locus. More individuals would provide extra information on the crossover and improve the resolution of the map and provide a benefit for the reference assembly improvement.

By comparing the genetic distance and unique markers in each linkage group, the density of the Mozambique map in this study is on the same level as that of the India map, with unique loci numbers that are approximately 2200 (Baranski et al., 2014). In general, the integration of different genetic maps is performed with common markers (Holtz et al., 2017). With the reference assembly as an intermediary, genetic maps that are constructed by different kinds of markers could be compared and integrated (Tang et al., 2015; Sutherland et al., 2016). However, the quality of our reference assembly is poor, and only half of the sequences from the India map can be downloaded from the database with the corresponding relation of linkage groups between the India map and Mozambique map not being established due to inadequate common scaffolds between the maps. For an India map, only 2114 mRNA sequences that were assigned to 1422 unique loci can be downloaded from the database of GenBank. These mRNA sequences were mapped to the reference assembly (unpublished) with the LAST program [30]. The corresponding relation of the linkage groups between these two maps was rebuilt with ALLMAPS (Tang et al., 2015). However, only 164 scaffolds were supported by at least two mRNA sequences, and 50 scaffolds were supported by the two maps, which only account for 0.5% of the scaffolds in number.

Various experiments have confirmed that the sex of the black tiger shrimp is determined by a WZ–ZZ chromosomal system (Benzie et al., 2001; Li et al., 2003; Staelens et al., 2008; Robinson et al., 2014). Our result also supports this conclusion. Even an approximately equal number of SNPs in the sex QTL interval shows the heterozygote favor, and the phased genotypes favor a female over male heterogamete. At the site X262, 93 out of 98 individuals support the WZ–ZZ system for which the segregation pattern in the female parent is associated with the sex determination. Sex determination in shrimp is confusing, as there has been no confirmed master sex-determining gene (Chandler et al., 2017). It was reported that the master sex-determining gene appears to be variable among different strains or populations in insects (Biedler and Tu, 2016) and in fish (Wilson et al., 2014). Two independent studies identified only one interval that contains the sex QTL in the black tiger shrimp (Staelens et al., 2008; Robinson et al., 2014). In this study, we compared the previous sex loci and our sex QTL, and the sequence alignment supports that these three loci are the same loci. The current black tiger shrimp is supposed to have ancestral origins of the Gondwana supercontinent (Waqairatu et al., 2012) and to have evolved through continual drift, ice age events, and environment adaption. Our evidence hints that the sex QTL may be the same in this species and provides the foundation for mapping the master sex-determining gene in the future. We found the gene SOX2 located in the sex QTL interval, which has been reported to be necessary for the normal development and function of the hypothalamo-pituitary and reproductive axes in humans and in mice (Kelberman et al., 2006), and also supposed to be essential in spermatogenesis and testis development in *Chlamys farreri* (Liang et al., 2019). However, the actual sex-determining gene is far from being discovered, with the fact that the sex determining varies in gene and form, such as piRNA in silkworms and alternative splicing in *Drosophila melanogaster* (Biedler and Tu, 2016) and SOX2 is located far from the supposed master sex-determining gene.

One of the applications of genetic markers is in sex identification, especially in fish (Mei and Gui, 2015). We also confirmed that the previously published sex-linked segment could also be used in our population. The segregation pattern only can be tested using polypropylene gel electrophoresis or capillary electrophoresis, which is time and cost consuming. A specific marker that could be tested in agarose gel electrophoresis needs to be developed in the future.

## Conclusion

Restriction-site associated DNA sequencing was applied to construct a high-density genetic linkage map for black tiger shrimp in this study, and our result supports the WZ–ZZ system. The sex QTL was located, and this locus was demonstrated to be the same as the loci in Mozambique, India, and Hawaiian populations.

## Ethics Statement

This study was carried out in accordance with the recommendations of the Animal Care and Use Committee of South China Sea Fisheries Research Institute, Chinese Academy of Fishery Sciences. The protocol was approved by the Animal Care and Use Committee of South China Sea Fisheries Research Institute, Chinese Academy of Fishery Sciences.

## Author Contributions

D-CZ conceived and designed this work. Y-HX and NZ executed the experiments. LG analyzed the data and wrote the manuscript. F-LZ, J-HH, B-SL, and S-GJ helped in the execution of some experiments. All authors discussed the results of the manuscript, reviewed the manuscript, and read and approved the final manuscript.

## Conflict of Interest Statement

Y-HX was employed by the company Biomarker Technologies Corporation. The remaining authors declare that the research was conducted in the absence of any commercial or financial relationships that could be construed as a potential conflict of interest.

## Footnotes

1 http://www.minitab.com

## Supplementary Material

TABLE S1 The pedigree information for the mapping family.

TABLE S2 The genotypes in Hapmap format for the 6524 SNPs.

TABLE S3 The SNPs in the constructed genetic map. The flanking sequences are provided.

TABLE S4 The genes located in the scaffolds containing the SNPs with the highest LOD in QTL mapping.
